# A correlation study of the relationships between nonalcoholic fatty liver disease and serum triglyceride concentration after an oral fat tolerance test

**DOI:** 10.1186/s12944-021-01483-z

**Published:** 2021-05-25

**Authors:** Xiaoyu Hou, Yunpeng Guan, Yong Tang, An Song, Jiajun Zhao, Luping Ren, Shuchun Chen, Limin Wei, Huijuan Ma, Guangyao Song

**Affiliations:** 1 Department of Internal Medicine, Hebei Medical University, Shijiazhuang, Hebei China; 2 Department of Endocrinology, Hebei General Hospital, 348, Heping West Road, Shijiazhuang, Hebei 050051 People’s Republic of China; 3 Key laboratory of Endocrinology, Ministry of Health, Department of Endocrinology, Peking Union Medical College Hospital, Peking Union Medical College, Chinese Academy of Medical Sciences, Beijing, China; 4 Department of Endocrinology and Metabolism, Shandong Provincial Hospital Affiliated to Shandong University, Jinan, Shandong China

**Keywords:** Nonalcoholic fatty liver disease, Postprandial lipidemia, Triglyceride, Oral fat tolerance test, Dyslipidemia, Clinical study

## Abstract

**Background:**

Nonalcoholic fatty liver disease (NAFLD) has become one of the most common chronic liver diseases worldwide. Triglyceride (TG) accumulation is central to NAFLD development. People now spend most of their day in the postprandial state, and the measurement of postprandial blood lipid concentration can make up for the lack of simple detection of fasting blood lipids. Postprandial triglyceride (PTG) is commonly used as a surrogate for postprandial blood lipid concentrations, and many studies have shown that PTG is a risk factor for NAFLD. The aim of the present study was to investigate the relationship between PTG concentration during oral fat tolerance testing (OFTT) and NAFLD.

**Methods:**

A total of 472 Chinese adults, aged 25 to 65 years, were enrolled in the study. All the participants underwent OFTT. The serum concentrations of TG and other lipids were measured, and their relationships with NAFLD were analyzed.

**Results:**

Of the 472 participants, 155 were diagnosed with NAFLD. The fasting and postprandial TG concentrations of the participants with NAFLD were higher than those of healthy participants (*P* < 0.05). The TG concentrations of the healthy participants peaked 4 h postprandially, whereas those of the participants with NAFLD peaked 6 h postprandially and reached higher peak values. Postprandial TG concentration was significantly associated with a higher risk of NAFLD.

**Conclusions:**

High PTG is positively related to a higher risk of NAFLD, and the PTG concentrations of patients with NAFLD are higher than in healthy individuals, with a delayed peak. Therefore, 4-h PTG may represent a potential marker of NAFLD.

**Trial registration:**

ChiCTR1800019514.

**Supplementary Information:**

The online version contains supplementary material available at 10.1186/s12944-021-01483-z.

## Background

Nonalcoholic fatty liver disease (NAFLD) is the most common liver disease worldwide. It is a clinicopathological syndrome that is characterized by excessive lipid deposition in hepatocytes, and can progress from nonalcoholic fatty liver to nonalcoholic steatohepatitis (NASH) and more severe diseases, which are characterized by hepatocellular injury and fibrosis, including cirrhosis and hepatocellular carcinoma [1, 2]. The proportion of cases of hepatocellular carcinoma that are caused by NAFLD has been increasing rapidly worldwide since 2000, and NAFLD has become the most frequent reason for liver transplantation in the USA [3–5]. Thus, the early identification of people who are more susceptible to NAFLD and the identification of patients in the early stages of NAFLD are of great importance for the prevention and treatment of NAFLD.

The exact mechanism of development of NAFLD is as yet unclear, multiple factors such as insulin resistance, nutritional factors, together with genetic predisposition, have some impacts on the development of NAFLD. In insulin resistant states, the insulin-induced suppression of adipose tissue lipolysis is impaired and the influx of free fatty acids into the liver increases [6]. Triglyceride (TG) is the main form of fat that accumulates in the liver of patients with NAFLD, and the TG content increases alongside lipotoxicity and liver injury. NAFLD is associated with a highly atherogenic lipoprotein profile, characterized by high serum TG, low-density lipoprotein (LDL-C), and apolipoprotein B (ApoB) concentrations and a low HDL-C concentration [7, 8]. In addition, previous studies have shown that hypertriglyceridemia is one of the risk factors for NAFLD [9], and that the prevalence and degree of hypertriglyceridemia significantly correlate with the severity of NAFLD [10, 11].

The fasting lipid profile of patients is most commonly assessed in clinical practice. However, people spend most of their day in the postprandial state TG is mostly affected by diet. Therefore, postprandial triglyceride (PTG) concentration is now commonly used as a surrogate for postprandial lipid status.

Recent studies have shown that dietary composition affects postprandial metabolism [12, 13]. A comparison of the effects of a Western-style high-fat diet, a Western-style high-carbohydrate diet, and a Mediterranean diet on lipid and glucose metabolism showed that energy-rich diets are associated with hyperglycemia, hyperlipidemia, an inflammatory response, and low concentrations of antioxidants [12]. Another study showed that meals that are high in saturated fat and carbohydrate affect the postprandial lipidemia of men with high waist circumference (WC) [13]. However, although various studies have shown a role for postprandial lipidemia, the definition of hypertriglyceridemia varies in different guidelines [14, 15]. A joint consensus statement from the European Atherosclerosis Society (EAS) and European Federation of Clinical Chemistry and Laboratory Medicine stated that the non-fasting TG concentration in patients with a fasting TG < 1.7 mmol/L should be < 2.0 mmol/L (175 mg/dL) [16]. In addition, a Greek expert panel statement defined TG concentrations ≤2.5 mmol/L (220 mg/dL) at all the time-points of an oral fat tolerance test (OFTT) as a desirable postprandial response [17]. Therefore, to further explore the relationship between hypertriglyceridemia and the metabolic abnormalities associated with NAFLD, it is important to establish a standardized OFTT and further define the relationship between PTG concentration and NAFLD.

The aim of the present study was to characterize the relationship between PTG concentration and the incidence of NAFLD by measuring the PTG concentrations during an OFTT, to evaluate the role of postprandial lipidemia in NAFLD.

## Methods

### Study sample

Four hundred seventy-two volunteers were randomly recruited at the Endocrinology Department of Hebei General Hospital, China, between May 2018 and December 2019. The study complied with the principles of the Declaration of Helsinki, the protocol was approved by the ethics committee of Hebei General Hospital, and all the participants gave their written informed consent. The study has been registered with the China Clinical Trial Registry (registration number: ChiCTR1800019514, registration date: November 15, 2018. http://www.chictr.org.cn/index.aspx).

### Exclusion criteria

Vegetarians; patients with malignant tumors, CVD, diabetes mellitus, thyroid dysfunction, kidney disease, hematologic disease, infectious disease, or psychiatric disorders; individuals who had experienced stroke or had been pregnant in the preceding 3 months; those who were taking drugs that influence lipid metabolism or inflammation, including fish oil, contraceptives, hormones, β receptor blockers, or diuretics; and patients who were under antidiabetic therapies and those who had experienced serious infection, surgery, trauma, or a body mass change of > 3 kg were excluded. After providing their written informed consent, physical examination and oral glucose tolerance testing (OGTT) were performed in all the participants. According to the results of OGTT, diabetic patients were excluded(volunteers whose fasting blood glucose(FBG) ≥ 7.0 mmol/L with or without 2-h blood glucose≥11.1 mmol/L were diagnosed as diabetic patients.).

All the participants also underwent OFTT, as described below.

### Oral fat tolerance testing

All the participants were asked to stick to their normal diet for 1 week before commencing the study. They were studied at Hebei General Hospital after a 10-h overnight fast. Venous blood was collected in the morning, and then the participants consumed a high-fat meal within the following 10 min. The high-fat meal was prepared by professional dieticians, and provided 1500 kcal in total, with fat, protein, and carbohydrate contents of 60, 20, and 20%, respectively. Blood samples were collected 2 h, 4 h, 6 h, 8 h, and 10 h after the consumption of the high-fat meal and the collected serum samples were stored at − 80 °C (Haier MDR-382E, China). During this 10-h period, the participants were allowed to drink only water and prohibited from smoking, or eating. Strenuous exercise was also prohibited; only slow walking was permitted.

### Measurement of anthropometric, clinical, and biochemical parameters

The FBG, apolipoprotein A1 (ApoA1), ApoB concentrations, and the total cholesterol (TC), TG, HDL-C, and LDL-C concentrations in the fasting state and 2 h, 4 h, 6 h, 8 h, and 10 h after the consumption of a high-fat meal were measured by laboratory technicians in the Physical Examination Center of Hebei General hospital using a Hitachi 7600 automatic biochemical analyzer (Hitachi Instruments Ltd., Tokyo, Japan). Fasting plasma insulin (FINS) concentration was measured using a chemiluminescence method in the Nuclear Medicine Department of Hebei General hospital. Homeostasis model assessment of insulin resistance (HOMA-IR) was calculated using the FINS and FBG concentrations by dividing insulin (μU/ml) and glucose (μmol/L) by 22.5 [18] (HOMA-IR = FBG ((mmol/L) × FINS (mIU/L)/22.5). Body mass index (BMI) was calculated as body mass divided by height squared (kg/m^2^).

Body mass, height, WC, systolic blood pressure (SBP), and diastolic blood pressure (DBP) were measured by trained professionals.

### Diagnosis of nonalcoholic fatty liver disease

Abdominal ultrasonography was performed by a specialist technologist in the Physical Examination Center of Hebei General hospital. A diagnosis of NAFLD was made according to The Guidelines for the Prevention and Treatment of Nonalcoholic Fatty Liver Disease (2018 update) [19] and the position statement on NAFLD/NASH that was derived from the European Association for the Study of the Liver 2009 special conference [20]. NAFLD can be diagnosed by imaging or histological findings of fatty liver, with the exclusion of other causes of liver steatosis (for example, viral, autoimmune, genetic, and use of drugs) and alcohol abuse (consumption of > 30 g/day in men and > 20 g/day in women) [20].

Ultrasonographic examinations were performed by a specialist technologist. Diffuse fatty liver was diagnosed if two of the following were present: (1) diffuse enhancement of the near-field echo of the liver (“bright liver”) and far-field echo attenuation; (2) unclear intrahepatic duct structure; (3) gradual attenuation of the far-field echo; and (4) mild-to-moderate hepatomegaly with a blunt leading edge.

### Statistical analysis

SPSS 21.0 software (IBM Inc., Armonk, NY, USA) was used for the statistical analysis. Numerical data were tested for normality using the Shapiro-Wilk test. Normally distributed data are expressed as mean ± standard deviation and non-normally distributed data are expressed as median and interquartile range. The independent sample *t*-test was used to compare data between two groups. Pearson’s chi-square test was used to compare the prevalence of NAFLD among the groups. Single-factor ANOVA was used to compare data among three groups. Binary logistic regression analysis, with NAFLD as the dependent variable, was used to determine the influence of each parameter on the prevalence of NAFLD. Statistical significance was accepted when *P* < 0.05.

## Results

All the participants consumed the high-fat meal during the OFTT and it was well tolerated.

### Basic characteristics

There were 472 participants in the present study, of whom 224 were male and 248 were female. The mean age of the men was 45 ± 13 years and that of the women was 44 ± 13 years (Table 1).

**Table 1:** Basic characters of the participants

Group	Total (*n* = 472)	Con (*n* = 317)	NAFLD (*n* = 155)	*P*
Age (Year)	44 ± 13	43 ± 14	47 ± 11^**##**^	< 0.001
Sex (Male/Female)	224/248	139/178	85/70	0.992
BMI (kg/m^2^)	25.93 ± 4.05	24.45 ± 3.25	28.97 ± 3.83^**##**^	< 0.001
SBP (mmHg)	127 ± 15	124 ± 15	133 ± 14^**##**^	< 0.001
DBP (mmHg)	78 ± 10	77 ± 9	83 ± 10^**##**^	< 0.001
WC (cm)	87.9 ± 11.9	83.9 ± 10.9	96.1 ± 9.4^**##**^	< 0.001
FBG (mmol/L)	5.41 (5.07,5.87)	5.29 (4.97,5.61)	5.78 (5.38,6.46) ^**##**^	< 0.001
FINS (mmol/L)	10.65 (7.35,15.24)	8.89 (6.43,11.84)	16.15 (11.19,19.90) ^**##**^	< 0.001
HOMA-IR	2.60 (1.70,3.82)	2.09 (1.47,2.95)	4.12 (3.01,5.71) ^**##**^	< 0.001
TC (mmol/L)	4.72 ± 1.01	4.59 ± 0.96	5.00 ± 1.06^**##**^	< 0.001
TG (mmol/L)	1.66 ± 1.31	1.34 ± 1.07	2.32 ± 1.50^**##**^	< 0.001
HDL-C(mmol/L)	1.25 ± 0.28	1.30 ± 0.29	1.14 ± 0.22^**##**^	< 0.001
LDL-C(mmol/L)	2.98 ± 0.73	2.85 ± 0.69	3.23 ± 0.74^**##**^	< 0.001
ApoA1(g/L)	1.40 (1.24,1.56)	1.42 (1.27,1.60)	1.34 (1.20,1.51) ^**#**^	=0.001
ApoB(g/L)	0.79 ± 0.22	0.75 ± 0.20	0.88 ± 0.23^**##**^	< 0.001
ApoA1/ApoB	1.78 (1.48,2.22)	1.94 (1.61,2.36)	1.57 (1.28,1.86) ^**##**^	< 0.001

Data are mean ± standard deviation or median (interquartile range)

*Abbreviations*: *BMI* body mass index, *WC* waist circumference, *SBP* systolic blood pressure, *DBP* diastolic blood pressure, *FBG* fasting blood glucose, *FINS* fasting plasma insulin concentration, *HOMA-IR* homeostasis model assessment of insulin resistance, *TC* total cholesterol, *TG* triglyceride, *HDL-C* high-density lipoprotein-cholesterol, *LDL-C* low-density lipoprotein-cholesterol, *ApoA1* apolipoprotein A1, *ApoB* apolipoprotein B

^#^ *P* < 0.05, compared with the Con group. ^##^*P* < 0.01, compared with the Con group

OGTT was used to exclude diabetic patients (Table S1).

The participants were allocated to the two groups according to their hepatic ultrasonographic findings. The fasting BMI, WC, SBP, DBP, FBG, FINS, TC, TG, LDL-C, ApoB, and HOMA-IR of the NAFLD group were higher than those of the control group (all *P* < 0.001). However, the HDL-C and ApoA1 concentrations and the ApoA1/ApoB ratio of the NAFLD group were lower than those of the control group (*P* < 0.001, *P* = 0.001, and *P* < 0.001, respectively) (Table 1).

The differences in the fasting BMI, FBG, FINS, HOMA-IR, TC, TG, HDL-C, LDL-C, ApoA1, ApoB, and ApoA1/ApoB of the NAFLD and control groups were affected by sex. The fasting BMI, FBG, FINS, and HOMA-IR of the male and female participants with NAFLD were higher than those of the same sex in the control group (*P* < 0.001). In addition, the fasting TC, TG, and LDL-C concentrations of the NAFLD group were higher than those of the control group among participants of the same sex, but the HDL-C concentration was lower than that of the control group (all *P* < 0.05) (Table 2).

**Table 2:** Comparison of anthropometric, clinical, and fasting metabolic parameters in participants of each sex in the NAFLD and control groups

	Con		NAFLD	
	Male(*n* = 139)	Female(*n* = 178)	Male(*n* = 85)	Female(*n* = 70)
Age (Year)	45 ± 14	42 ± 13	44 ± 12	51 ± 10
BMI (kg/m^2^)	25.57 ± 3.17	23.58 ± 3.04	29.29 ± 3.55^**##**^	28.58 ± 4.14^**##**^
FBG (mmol/L)	5.40 (5.07,5.79)	5.22 (4.93,5.54)	5.79 (5.40,6.47) ^**##**^	5.78 (5.37,6.49) ^**##**^
FINS (mmol/L)	9.40 (6.48,12.34)	8.63 (6.14,11.60)	16.40 (11.17,20.57) ^**##**^	15.33 (11.45,19.34) ^**##**^
HOMA-IR	2.09 (1.56,3.07)	2.07 (1.39,2.76)	4.28 (3.04,5.86) ^**##**^	4.07 (2.97,5.67) ^**##**^
TC (mmol/L)	4.43 ± 0.86	4.71 ± 1.02	4.82 ± 0.99^**#**^	5.21 ± 1.10^**#**^
TG (mmol/L)	0.99 ± 0.30	0.86 ± 0.26	1.57 ± 0.48^**##**^	1.78 ± 0.77^**##**^
HDL-C (mmol/L)	1.18 ± 0.26	1.39 ± 0.29	1.06 ± 0.16^**##**^	1.24 ± 0.24^**##**^
LDL-C (mmol/L)	2.79 ± 0.60	2.90 ± 0.76	3.14 ± 0.71^**##**^	3.34 ± 0.78^**##**^

Data are mean ± standard deviation or median (interquartile range)

*Abbreviations*: *Con* control group, *BMI* body mass index, *FBG* fasting blood glucose, *FINS* fasting plasma insulin concentration, *HOMA-IR* homeostasis model assessment of insulin resistance, *TC* total cholesterol, *TG* triglyceride, *HDL-C* high-density lipoprotein-cholesterol, *LDL-C* low-density lipoprotein-cholesterol

^#^ *P* < 0.05, compared with the Con group. ^##^ *P* < 0.01, compared with the Con group

### Circulating lipid concentrations at time points during the OFTT

The TC, TG, and LDL-C concentrations in the fasting state, and 2 h, 4 h, 6 h, 8 h, and 10 h after the start of the OFTT were higher in the NAFLD group than in the control group, whereas the HDL-C concentration was lower (*P* < 0.001). Because lipid metabolism is known to differ according to sex, the circulating lipid concentrations at time points during the OFTT were further analyzed. Among the male participants, patients with NAFLD had higher concentrations of TC, TG, and LDL-C, and a lower HDL-C concentration than those of the control group. The TC concentrations in the fasting state, and 2 h, 4 h, 6 h, 8 h, and 10 h after the high-fat meal were significantly higher than those of the control group (*P* < 0.05, *P* < 0.05, *P* < 0.001, *P* < 0.001, *P* < 0.001, and *P* < 0.001, respectively). The female patients with NAFLD also had significantly higher TC, TG, and LDL-C, and lower HDL-C concentrations (all *P* < 0.001) than those of the control group (Table 3).

**Table 3:** Lipid metabolism parameters during the oral fat tolerance test

		0 h	2 h	4 h	6 h	8 h	10 h
Con	TC	4.59 ± 0.96	4.56 ± 0.94	4.55 ± 0.96	4.70 ± 0.98	4.75 ± 0.99	4.70 ± 0.95
	TG	1.34 ± 1.07	2.20 ± 1.27	2.65 ± 1.75	2.59 ± 2.03	2.33 ± 2.04	1.57 ± 1.62
	HDL-C	1.30 ± 0.29	1.30 ± 0.29	1.24 ± 0.28	1.24 ± 0.29	1.29 ± 0.30	1.28 ± 0.29
	LDL-C	2.85 ± 0.69	2.78 ± 0.67	2.72 ± 0.65	2.80 ± 0.67	2.84 ± 0.68	2.88 ± 0.67
Con (male)	TC	4.42 ± 0.86	4.42 ± 0.87	4.43 ± 0.89	4.59 ± 0.89	4.63 ± 0.88	4.61 ± 0.90
	TG	1.56 ± 1.22	2.47 ± 1.45	3.11 ± 1.98	3.15 ± 2.30	2.92 ± 2.41	1.98 ± 2.06
	HDL-C	1.18 ± 0.26	1.19 ± 0.27	1.13 ± 0.26	1.14 ± 0.27	1.17 ± 0.28	1.17 ± 0.27
	LDL-C	2.79 ± 0.60	2.73 ± 0.60	2.68 ± 0.58	2.76 ± 0.57	2.80 ± 0.58	2.86 ± 0.61
Con (female)	TC	4.71 ± 1.02	4.67 ± 0.97	4.65 ± 1.00	4.78 ± 1.05	4.83 ± 1.06	4.77 ± 0.98
	TG	1.17 ± 0.90	1.99 ± 1.06	2.29 ± 1.44	2.15 ± 1.67	1.87 ± 1.55	1.26 ± 1.08
	HDL-C	1.39 ± 0.29	1.39 ± 0.28	1.32 ± 0.27	1.33 ± 0.28	1.38 ± 0.28	1.37 ± 0.28
	LDL-C	2.90 ± 0.76	2.83 ± 0.72	2.76 ± 0.70	2.83 ± 0.74	2.88 ± 0.75	2.89 ± 0.72
NAFLD	TC	5.00 ± 1.06^##^	4.96 ± 1.03^##^	5.05 ± 1.07^##^	5.22 ± 1.09^##^	5.24 ± 1.10^##^	5.21 ± 1.20^##^
	TG	2.32 ± 1.50^##^	3.47 ± 1.59^##^	4.32 ± 2.04^##^	4.50 ± 2.54^##^	4.25 ± 2.61^##^	2.96 ± 2.45^##^
	HDL-C	1.14 ± 0.22^##^	1.15 ± 0.22^##^	1.09 ± 0.21^##^	1.07 ± 0.22^##^	1.11 ± 0.22^##^	1.10 ± 0.22^##^
	LDL-C	3.23 ± 0.74^##^	3.14 ± 0.72^##^	3.09 ± 0.70^##^	3.16 ± 0.69^##^	3.20 ± 0.70^##^	3.28 ± 0.75^##^
NAFLD (male)	TC	4.82 ± 0.99^&^	4.78 ± 0.94^&^	4.91 ± 1.03^&&^	5.08 ± 1.05^&&^	5.10 ± 1.07^&^	5.08 ± 1.20^&^
	TG	2.54 ± 1.73^&&^	3.73 ± 1.67^&&^	4.60 ± 2.07^&&^	4.81 ± 2.50^&&^	4.52 ± 2.63^&&^	3.07 ± 2.45^&&^
	HDL-C	1.06 ± 0.16^&&^	1.07 ± 0.17^&&^	1.02 ± 0.17^&^	1.01 ± 0.16^&&^	1.04 ± 0.18^&&^	1.03 ± 0.17^&&^
	LDL-C	3.14 ± 0.71^&&^	3.05 ± 0.69^&&^	3.02 ± 0.68^&&^	3.08 ± 0.66^&&^	3.12 ± 0.67^&&^	3.22 ± 0.72^&&^
NAFLD (female)	TC	5.21 ± 1.10^%^	5.18 ± 1.09^%%^	5.22 ± 1.11^%%^	5.39 ± 1.12^%%^	5.41 ± 1.13^%%^	5.36 ± 1.19^%%^
	TG	2.04 ± 1.13^%%^	3.16 ± 1.44^%%^	3.98 ± 1.97^%%^	4.11 ± 2.54^%%^	3.92 ± 2.56^%%^	2.83 ± 2.47^%%^
	HDL-C	1.24 ± 0.24^%%^	1.25 ± 0.23^%%^	1.17 ± 0.23^%%^	1.15 ± 0.25^%%^	1.19 ± 0.24^%%^	1.18 ± 0.24^%%^
	LDL-C	3.34 ± 0.78^%%^	3.25 ± 0.75^%%^	3.17 ± 0.72^%%^	3.26 ± 0.73^%%^	3.29 ± 0.73^%%^	3.34 ± 0.79^%%^

Data are mean ± standard deviation

*Abbreviations*: *Con* control group, *NAFLD* Nonalcoholic fatty liver disease, *TC* total cholesterol, *TG* triglyceride, *HDL-C* high-density lipoprotein-cholesterol, *LDL-C* low-density lipoprotein-cholesterol

^#^ *P* < 0.05, compared with the control group. ^##^ *P* < 0.01, compared with the control group

^*&*^ *P* < 0.05, compared with the male control group. ^*&&*^ *P* < 0.01, compared with the male control group

^*%*^ *P* < 0.05, compared with the female control group. ^*%%*^ *P* < 0.01, compared with the female control group

### TG peak concentration and timing

**Fig. 1:**
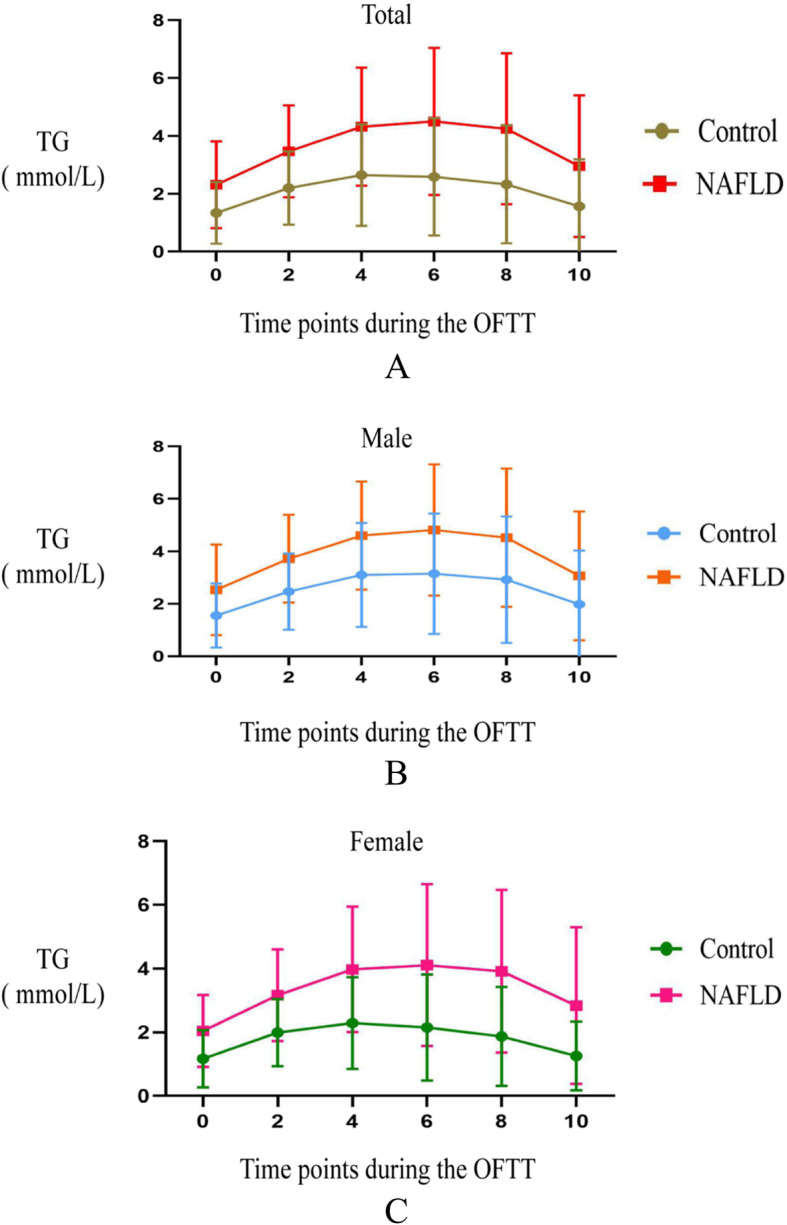
Triglyceride concentrations at time points during oral fat tolerance test; A-Total; B-Female; C-Male. TG-triglyceride; NAFLD- nonalcoholic fatty liver disease

A graph of the TG concentration at time points during the OFTT was constructed and analyzed according to sex. The TG concentration in each group increased gradually after the ingestion of the high-fat meal during the OFTT. Regardless of sex, the PTG of the control group peaked 4 h postprandially while the TG of the NAFLD group peaked 6 h postprandially (Fig. 1a-c).

### Comparisons of parameters among groups of participants with differing fasting TG concentration

According to their fasting TG concentration, the participants were allocated to one of two groups: those with a fasting TG concentration ≤ 1.7 mmol/L were placed in a normal-TG group (NFTG group) and those with TG > 1.7 mmol/L were placed in a high fasting TG group (HFTG group). The fasting BMI, FBG, FINS, and HOMA-IR of the HFTG group were higher than those of the NFTG group (*P* < 0.001). The incidence of NAFLD in the participants as a whole was 32.8%. Among participants who had a normal fasting TG, the incidence of NAFLD was 19.9%, whereas it was 60.7% in the high-TG group. Pearson’s chi-square test showed that the incidence of NAFLD between the two groups significantly differed (*P* < 0.001) (Table 4).

**Table 4:** Comparison of the characteristics of the NFTG and HFTG

	Total	NFTG	HFTG	*P*
	n = 472	*n* = 322	*n* = 150	
Age	44 ± 13	43 ± 14	47 ± 11	0.176
Sex(male/female)	224/248	133/189	91/59	0.084
BMI	25.93 ± 4.05	24.94 ± 3.76	28.06 ± 3.85^**^	< 0.001
FBG	5.41 (5.07,5.87)	5.32 (5.00,5.73)	5.60 (5.34,6.31) ^**^	< 0.001
FINS	10.65 (7.35,15.24)	9.55 (6.59,12.72)	13.85 (10.20,18.83) ^**^	< 0.001
HOMA-IR	2.60 (1.70,3.82)	2.24 (1.52,3.11)	3.70 (2.60,5.07) ^**^	< 0.001
TC	4.72 ± 1.01	4.50 ± 0.93	5.19 ± 1.02^**^	< 0.001
TG	1.66 ± 1.31	1.04 ± 0.32	2.98 ± 1.62^**^	< 0.001
HDL-C	1.25 ± 0.28	1.30 ± 0.28	1.14 ± 0.24^**^	< 0.001
LDL-C	2.98 ± 0.73	2.82 ± 0.69	3.32 ± 0.70^**^	< 0.001
NAFLD	155 (32.8%)	64 (19.9%)	91 (60.7%) ^**^	< 0.001
Non-NAFLD	317 (67.2%)	258 (80.1%)	59 (39.3%)	< 0.001

Data are mean ± standard deviation, median (interquartile range), or number (percentage)

*Abbreviations NFTG* normal fasting triglyceride group, *HFTG* High fasting triglyceride group, *BMI* body mass index, *FBG* fasting blood glucose, *FINS* fasting plasma insulin concentration, *HOMA-IR* homeostasis model assessment of insulin resistance, *TC* total cholesterol, *TG* triglyceride, *HDL-C* high-density lipoprotein-cholesterol, *LDL-C* low-density lipoprotein-cholesterol, *NAFLD* nonalcoholic fatty liver disease

^*^ *P* < 0.05, compared with the NFT group. ^**^ *P* < 0.01, compared with the NFT group

### Factors associated with NAFLD

**Fig. 2:**
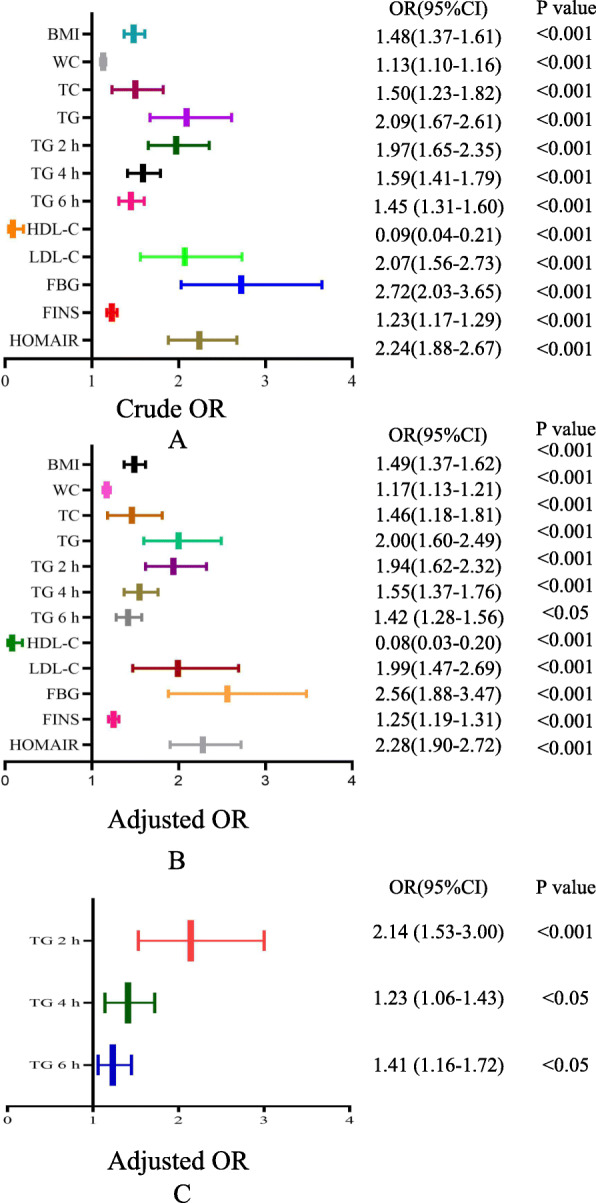
Odds ratio of factors influencing NAFLD. A-Crude OR; B-Adjusted age and gender; C- Adjusted age, sex, fasting triglycerides and BMI; OR: odds ratio; 95%CI: 95% Confidence Interval; TG 2 h: triglyceride 2 h postprandially; TG 4 h: triglyceride 4 h postprandially

The relationships of fasting BMI, WC, TC, TG, FBG, FINS, HOMA-IR, HDL-C, LDL-C, and fat load with NAFLD were determined using binary logistic regression analysis, and the factors that might predispose or protect the participants from NAFLD were further analyzed using forest plots. The parameters that were shown to be associated with the prevalence of NAFLD were BMI, WC, TC, TG, 2-h PTG (TG 2 h), 4-h PTG (TG 4 h), 6-h PTG (TG 6 h), HDL-C, LDL-C, HOMA-IR, FBG, and FINS (BMI: B 0.39, odds ratio [OR] 1.48, 95% confidence interval [CI] 1.37–1.61; WC: B 0.13, OR 1.13, 95% CI 1.10–1.16; TC: B 0.40, OR 1.50, 95% CI 1.23–1.82; TG: B 0.74, OR 2.09, 95% CI 1.67–2.61; TG 2 h: B 0.68, OR 1.97, 95% CI 1.65–2.35; TG 4 h: B 0.46, OR 1.59, 95% CI 1.41–1.79; TG 6 h: B 0.37, OR 1.45, 95% CI 1.31–1.60; HDL-C: B − 2.37, OR 0.09, 95% CI 0.04–0.21; LDL-C: B 0.73, OR 2.07, 95% CI 1.56–2.73; FBG: B 1.00, OR 2.72, 95% CI 2.03–3.65; FINS: B 0.21, OR 1.23, 95% CI 1.17–1.29; HOMA-IR: B 0.81, OR 2.24, 95% CI 1.88–2.67; Table 5, Model 1, Fig. 2a).

**Table 5:** Odds ratio of impacting factors for NAFLD

Variables	Unstandardized Coefficient (B)	SE	Odds ratio (95% CI)	*P* value
**Model 1**				
BMI	0.39	0.04	1.48 (1.37–1.61)	< 0.001
WC	0.13	0.01	1.13 (1.10–1.16)	< 0.001
TC	0.40	0.10	1.50 (1.23–1.82)	< 0.001
TG	0.74	0.11	2.09 (1.67–2.61)	< 0.001
TG 2 h	0.68	0.09	1.97 (1.65–2.35)	< 0.001
TG 4 h	0.46	0.06	1.59 (1.41–1.79)	< 0.001
TG 6 h	0.37	0.05	1.45 (1.31–1.60)	< 0.001
HDL-C	−2.37	0.42	0.09 (0.04–0.21)	< 0.001
LDL-C	0.73	0.14	2.07 (1.56–2.73)	< 0.001
FBG	1.00	0.15	2.72 (2.03–3.65)	< 0.001
FINS	0.21	0.02	1.23 (1.17–1.29)	< 0.001
HOMA-IR	0.81	0.09	2.24 (1.88–2.67)	< 0.001
**Model 2**				
BMI	0.40	0.04	1.49 (1.37–1.62)	< 0.001
WC	0.15	0.02	1.17 (1.13–1.21)	< 0.001
TC	0.38	0.11	1.46 (1.18–1.81)	< 0.001
TG	0.69	0.11	2.00 (1.60–2.49)	< 0.001
TG 2 h	0.66	0.09	1.94 (1.62–2.32)	< 0.001
TG 4 h	0.44	0.06	1.55 (1.37–1.76)	< 0.001
TG 6 h	0.21	0.05	1.42 (1.28–1.56)	< 0.05
HDL-C	−2.51	0.46	0.08 (0.03–0.20)	< 0.001
LDL-C	0.69	0.15	1.99 (1.47–2.69)	< 0.001
FBG	0.94	0.16	2.56 (1.88–3.47)	< 0.001
FINS	0.22	0.03	1.25 (1.19–1.31)	< 0.001
HOMA-IR	0.82	0.09	2.28 (1.90–2.72)	< 0.001
**Model 3**				
TG 2 h	0.76	0.17	2.14 (1.53–3.00)	< 0.001
TG 4 h	0.34	0.10	1.41 (1.16–1.72)	< 0.05
TG 6 h	0.21	0.07	1.23 (1.06–1.43)	< 0.05

Model 1-Crude OR; Model 2- Adjusted age and gender; Model 3- Adjusted age, gender fasting triglyceride and BMI

*Abbreviations*: *BMI* body mass index, *WC* waist circumference, *FBG* fasting blood glucose, *FINS* fasting plasma insulin concentration, *HOMA-IR* homeostasis model assessment of insulin resistance, *TC* total cholesterol, *TG* triglyceride, *TG2 h* triglyceride 2 h postprandially, *TG4 h* triglyceride 4 h postprandially, *HDL-C* high-density lipoprotein-cholesterol, *LDL-C* low-density lipoprotein-cholesterol, *95% CI* 95% confidence interval

After adjustment for age and sex, the factors listed above were still associated with NAFLD (BMI: B 0.40, OR 1.49, 95% CI 1.37–1.62; WC: B, 0.15, OR 1.17, 95% CI 1.13–1.21; TC: B 0.38, OR 1.46, 95% CI 1.18–1.81; TG: B 0.69, OR 2.00, 95% CI 1.60–2.49; TG 2 h: B 0.66, OR 1.94, 95% CI 1.62–2.32; TG 4 h: B 0.44, OR 1.55, 95% CI 1.37–1.76; TG 6 h: B 0.21, OR 1.42, 95% CI 1.28–1.56; HDL-C: B − 2.51, OR 0.08, 95% CI 0.03–0.20; LDL-C: B 0.69, OR 1.99, 95% CI 1.47–2.69; FBG: B 0.94, OR 2.56, 95% CI 1.88–3.47; FINS: B 0.22, OR 1.25, 95% CI 1.19–1.31; HOMA-IR: B 0.82, OR 2.28, 95% CI 1.90–2.72; Table 5, Model 2, Fig. 2b). To exclude the effects of age, sex and fasting TG, the data were further adjusted, after which high PTG was still associated with a higher likelihood of having NAFLD (TG 2 h: B 0.63, OR 1.88, 95% CI 1.40–2.54; TG 4 h: B 0.32, OR 1.37, 95% CI 1.14–1.65; TG 6 h: B 0.21, OR 1.23, 95% CI 1.06–1.43; Table 5, Model 3, Fig. 2c).

### Characteristics of participants with differing levels of fat tolerance

The participants were allocated to three groups according to their fasting TG and 4-h PTG concentrations. A 4-h PTG concentration > 2.5 mmol/L was defined as a high postprandial TG (HTG) concentration [21]. Participants with normal fasting triglyceride and PTG concentrations were placed in the normal fat tolerance group (NFT), those with a normal fasting concentration but an HTG were placed in the impaired fat tolerance (IFT) group, and those with a high fasting TG concentration were placed in the fasting hypertriglyceridemia (FHT) group (Table 6).

**Table 6:** Characteristics of different fat tolerance groups

	NFT *n* = 192	IFT *n* = 130	FHT n = 150
Age	40 ± 13	48 ± 13^##^	47 ± 11^##^
Sex(male/female)	66/126	63/67	91/59
BMI	24.18 ± 3.68	26.07 ± 3.60^##^	28.06 ± 3.85^##**^
WC	82.43 ± 10.96	88.24 ± 11.32^##^	94.71 ± 9.79^##**^
FBG	5.32 ± 0.56	5.72 ± 1.36^#^	6.00 ± 1.29^##^
FINS	9.56 ± 5.20	12.18 ± 6.95^#^	15.95 ± 9.91^##*^
HOMA-IR	2.29 ± 1.36	3.22 ± 2.51^##^	4.33 ± 2.96^##*^
TC	4.34 ± 0.88	4.74 ± 0.95^##^	5.19 ± 1.02^##**^
TG	0.88 ± 0.26	1.28 ± 0.25^##^	2.98 ± 1.62^##**^
HDL-C	1.34 ± 0.29	1.23 ± 0.26^##^	1.14 ± 0.24^##*^
LDL-C	2.66 ± 0.65	3.05 ± 0.69^##^	3.32 ± 0.70^##**^
NAFLD	24 (12.5%)	40 (30.7%) ^##^	91 (60.6%) ^##**^

Data are mean ± standard deviation, median (interquartile range), or number (percentage)

*Abbreviations*: *BMI* body mass index, *WC* waist circumference, *FBG* fasting blood glucose, *FINS* fasting plasma insulin concentration, *HOMA-IR* homeostasis model assessment of insulin resistance, *TC* total cholesterol, *TG* triglyceride, *HDL-C* high-density lipoprotein-cholesterol, *LDL-C* low-density lipoprotein-cholesterol, *NAFLD* nonalcoholic fatty liver disease, *NFT* normal fat tolerance, *IFT* impaired fat tolerance, *FHT* fasting hypertriglyceridemia

^#^ *P* < 0.05, compared with the NFT group. ^##^ *P* < 0.01, compared with the NFT group. ^*^ *P* < 0.05, compared with the IFT group. ^**^ *P* < 0.01, compared with the IFT group

Furthermore, considering PTG peaked at 6 h after meal and 6-h PTG is a risk factor of NAFLD independent of age, sex and FTG, all the participants were allocated into different fat tolerance groups defined by FTG and 6-h PTG concentration (Table 7).

**Table 7:** Characteristics of different fat tolerance groups allocated according to FTG and 6 h PTG

	NFT *n* = 230	IFT *n* = 92	FHT *n* = 150
Age	41 ± 14	49 ± 12^##^	47 ± 11^##^
Sex(male/female)	82/148	51/41	91/59
BMI	24.4 ± 3.67	26.29 ± 3.65^##^	28.06 ± 3.85^##*^
WC	82.7 ± 11.52	89.96 ± 9.52^##^	94.71 ± 9.79^##^
FBG	5.36 ± 0.75	5.77 ± 1.38^#^	6.00 ± 1.29^##^
FINS	10.12 ± 5.7	11.86 ± 6.87^#^	15.95 ± 9.91^##**^
HOMA-IR	2.46 ± 1.62	3.15 ± 2.56^#^	4.33 ± 2.96^##**^
TC	4.38 ± 0.86	4.81 ± 1.01^#^	5.19 ± 1.02^##*^
TG	0.95 ± 0.29	1.28 ± 0.27^##^	2.98 ± 1.62^##**^
HDL-C	1.33 ± 0.28	1.22 ± 0.26^#^	1.14 ± 0.24^##^
LDL-C	2.69 ± 0.63	3.13 ± 0.73^##^	3.32 ± 0.7^##^
NAFLD	32 (13.9%)	32 (34.8%) ^##^	91 (60.6%) ^##**^

Data are mean ± standard deviation, or number (percentage)

^#^ *P* < 0.05, compared with the NFT group. ^##^ *P* < 0.01, compared with the NFT group. ^*^ *P* < 0.05, compared with the IFT group. ^**^ *P* < 0.01, compared with the IFT group

Although the criteria were different, across the three groups of different fat tolerance, fasting BMI, WC, FINS, HOMA-IR, TC, TG, and LDL-C all increased with decreasing fat tolerance, whereas HDL-C decreased with decreasing fat tolerance (*P* < 0.05). The age and FBG of the IFT and FHT groups were higher than those of the NFT group (*P* < 0.05), but there were no significant differences in age or FBG between the IFT and FHT groups. In addition, the incidence of NAFLD increased as the fat tolerance of the participants decreased. The incidence of NAFLD in the NFT group was relatively low, at 12.5%, whereas 30.7% of the participants in the IFT group had NAFLD, and the incidence of NAFLD in the FHT group was much higher, at 60.6% (fat tolerance was defined according to FTG and 4-h PTG). The differences among the three groups were significant (*P* < 0.001). Meanwhile, across the three groups of different fat tolerance defined according to FTG and 6-h PTG, the incidences of NAFLD in NFT, IFT and FHT groups were 13.9, 34.8 and 60.6%. The differences among the groups were significant at the same time (*P* < 0.001).

## Discussion

According to the results of the present study, NAFLD is very common in people of Han ethnicity, with a prevalence of 32.8%. By using binary logistic regression, BMI, WC, and TG were found to be associated with NAFLD, including TG after a fat load. After adjustment for age and sex, individuals with a high PTG remained more likely to have NAFLD.

By analogy with OGTT, the fasting TG and 4-h PTG concentrations during an OFTT were used as criteria to define fat tolerance, and all the participants were allocated to three groups of differing fat tolerance, according to their fasting TG and PTG concentrations. According to the Greek consensus [21], PTG after the consumption of the high-fat meal during the OFTT of > 2.5 mmol/L was considered as high. Participants who had a fasting TG concentration within the normal range but a PTG higher than the desirable concentration were found to have a higher incidence of NAFLD than those with normal fasting TG and PTG concentrations. Therefore, if only the fasting TG concentration is measured as the normal clinical routine, a diagnosis of NAFLD may be missed. Instead, the measurement of PTG may help to identify patients in the early stages of NAFLD, which may lead to better clinical outcomes.

In this study, we analyzed basic characters such as BMI, WC and the incidences of NAFLD in different fat tolerance groups separately defined by FTG and 4-h PTG or FTG and 6-h PTG. The results showed that, the incidence of NAFLD climbed when fat tolerance declined. Although PTG peaked at 6 h after meal, 4-h PTG is more convenient for clinical practice. Furthermore, the use of a 4-h postprandial value to define hypertriglyceridemia is consistent with practices reported by multiple authors in other countries [22–25]. A study that assessed the determinants of PTG in healthy young adults found a strong correlation between 4-h PTG and fasting TG, and therefore the authors came to the conclusion that 4-h PTG might be suitable as a replacement for fasting TG when this cannot easily be measured [26].

A meta-analysis conducted by Mihas et al. showed that a high TG concentration 4 h after a fat load is indicative of an excessive response [22]. The meta-analysis also suggested that a fat load of 70–79 g is optimal. However, the high-fat meal administered in the present study contained 100 g fat. Differences in the fat load may affect the postprandial lipid concentrations, as may the ethnicity of the participants. However, although the meta-analysis was only of studies of Caucasians and the participants in the present study were all Chinese, their TG concentrations also peaked at 4 h postprandially. To determine the most suitable time point for the assessment of postprandial lipids during an OFTT in Chinese people, further studies are required. In addition, the definition of PTG remains unclear. The criteria used in the present study was higher than that after a daily meal recommended by the EAS consensus statement [16] and that after a daily Chinese breakfast in overweight people [21] while lower than that in another Mexican study [27]. Disparities in the levels used in the various studies may be related to the differing ethnicity of the participants and differences in their standard dietary calorie and fat intake.

Participants with NAFLD were found to have higher BMI, WC, SBP, DBP, and HOMA-IR, which is consistent with previous studies. Obese but metabolically healthy individuals have been shown to be at a greater risk of NAFLD progression than metabolically healthy individuals of a normal weight [28]. In addition, WC is a risk factor for NAFLD because it is associated with IR and hypertension [29–31]. An analysis of the characteristics of participants with differing fasting TG concentrations showed that BMI is higher in individuals with high fasting TG concentrations. Furthermore, the study by Nogueira et al. showed that overweight and obesity are associated with a high prevalence of dyslipidemia [32], and another study showed that obese and insufficiently active male adolescents are more likely to have a high circulating TG concentration [33].

High TC, TG, and LDL-C concentrations are risk factors for CVD [34], whereas HDL-C has protective effects [35]. HDL-C is mainly involved in the reverse transport of cholesterol from extrahepatic tissues to the liver, but it can also inhibit the uptake of LDL-C by arterial smooth muscle cells and prevent the accumulation of cholesterol in cells. Therefore, HDL-C has anti-atherosclerotic effects and is often used as a marker of CVD risk [36]. In the present study, patients with NAFLD were found to have abnormal lipid metabolism, indicated by high concentrations of TC, TG, and LDL-C and a low concentration of HDL-C. ApoB regulates the secretion of very low-density lipoprotein, which is present at high circulating concentrations in patients with obesity, T2DM, and hypertriglyceridemia. Therefore, the high ApoB concentration in the NAFLD group is further evidence of dyslipidemia in these patients.

The TG concentration peaked later after a meal in the NAFLD group, which may imply that the meal-induced change in serum TG is larger in patients with NAFLD than in normal individuals. An impairment in TG metabolism is also suggested by the long period of time taken for the TG concentration to return to baseline following a high-fat meal.

Dyslipidemia is the principal etiologic factor in the development of NAFLD in non-diabetic patients [37]. A previous study showed that dyslipidemia is common in lean non-diabetic patients with NAFLD, with similar lipid profiles being identified in such patients to those who were overweight or obese [38]. The results of this study also suggested that high TG and low HDL-C concentrations may influence the development of NAFLD in non-diabetic patients.

### Study strength and limitations

The strengths of the present study are as follows. To the best of our knowledge, this was the first study to use a standardized OFTT in a large sample of Chinese patients to assess the relationship between PTG and NAFLD. In this study, 4-h PTG is significantly related to NAFLD. The 4-h time point after eating is consistent with the results of a previous study that showed a strong correlation between 4-h PTG and fasting TG [26].

Some limitations to the study should also be acknowledged. Abdominal ultrasound is not sensitive to diagnose fatty liver. Subjects with mild steatosis are possibly missed. Therefore, the conclusion of this study may be limited to some extent. The results of the present study cannot be extrapolated to other populations because it only included Chinese patients. In a clinical trial, it is difficult to match participants for their age and sex. However, analysis of the data for each sex showed that patients with NAFLD of both sexes have poorer fasting lipid profiles than those without. Furthermore, after adjustment for age and sex, 4-h PTG remained positively associated with a higher likelihood of having NAFLD.

## Conclusions

A high 4-h PTG concentration were found to be significantly and positively related to NAFLD. In addition, individuals with a high PTG have a higher prevalence of NAFLD. Thus, 4-h PTG may represent a potential marker of NAFLD. The results showed that the incidence of NAFLD in individuals with normal fasting TG but high PTG is high, at 30.7%. Therefore, the measurement of fasting TG alone may result in the misdiagnosis of some metabolically unhealthy patients who have normal fasting TG concentrations. NAFLD is asymptomatic in most patients, and therefore it remains a diagnosis of exclusion. Although liver biopsy is the gold-standard method of distinguishing the different stages of NAFLD, it is not always used in clinical practice because of its invasiveness. Therefore, early identification and treatment of NAFLD may become more feasible if PTG is measured. In addition, nutritional factors influence NAFLD, and PTG may be useful as a means of monitoring the efficacy of therapeutic interventions in patients with NAFLD.

## Supplementary Information

**Additional file 1: Table S1.** Oral glucose tolerance test results of participants with or without NAFLD

## Data Availability

The datasets generated in this study and the protocol are available on reasonable request. Please contact the corresponding author.

## Abbreviations

NAFLD: Nonalcoholic fatty liver disease
NASH: Nonalcoholic steatohepatitis
CVD: Chronic cardiovascular disease
BMI: Body mass index
SBP: Systolic blood pressure
DBP: Diastolic blood pressure
WC: Waist circumference
FBG: Fasting blood glucose
FINS: Fasting plasma insulin concentration
HOMA-IR: Homeostasis model assessment of insulin resistance
TC: Total cholesterol
TG: Triglyceride
HDL-C: High-density lipoprotein-cholesterol
LDL-C: Low-density lipoprotein-cholesterol
ApoA1: Apolipoprotein A1
ApoB: Apolipoprotein B
Con: Control group
NFTG: Normal fasting triglyceride group
HFTG: High fasting triglyceride group
NFT: Normal fat tolerance
IFT: Impaired fat tolerance
FHT: Fasting hypertriglyceridemia
TG 2 h: 2-h postprandial triglyceride
TG 4 h: 4-h postprandial triglyceride
95% CI: 95% confidence interval
