# Supplementary material for: A correlation study of the relationships between nonalcoholic fatty liver disease and serum triglyceride concentration after an oral fat tolerance test
Source: Lipids Health Dis. 2021 May 25;20:54. doi: 10.1186/s12944-021-01483-z (PMC8152134; doi:10.1186/s12944-021-01483-z)
Supplement: Supplementary file 1 — Additional file 1: Table S1. Oral glucose tolerance test results of participants with or without NAFLD [file 12944_2021_1483_MOESM1_ESM.docx]

**Supplementary materials**

Table S1 Oral glucose tolerance test results of participants with or without NAFLD

| Group | BG (mmol/L) | BG0.5 h (mmol/L) | BG1 h (mmol/L) | BG2 h (mmol/L) | BG3 h (mmol/L) |
| --- | --- | --- | --- | --- | --- |
| Total(n=472） | 5.17(4.83,5.61) | 8.92(7.78,10.03) | 8.21(6.31,10.52) | 6.12(5.13,8.155) | 4.28(3.69,5.28) |
| Con  (n=317) | 5.02(4.74,5.35) | 8.38(7.36,9.60) | 7.33(5.80,9.20) | 5.69(4.91,6.82) | 4.07(3.57,4.79) |
| Con (male)  (n=139) | 5.13(4.80,5.54) | 8.85(7.91,9.92) | 7.84(6.35,10.23) | 5.78(4.86,7.53) | 4.08(3.57,4.80) |
| Con (female)  (n=178) | 4.98(4.73,5.26) ^#^ | 8.07(7.11,9.23) ^##^ | 6.85(5.37,8.53) ^##^ | 5.68(4.98,6.72) | 4.06(3.57,4.80) |
| NAFLD  (n=155) | 5.55(5.16,6.39) | 9.87(8.83,11.60) | 10.26(8.41,13.20) | 7.90(6.07,10.94) | 5.02(4.03,6.62) |
| NAFLD (male)  (n=85) | 5.44(5.09,11.42) ^##^ | 9.66(8.57,12.87) ^#^ | 10.26(8.20,10.45) ^##^ | 7.58(5.90,6.32) ^##^ | 4.98(3.92,6.32) ^##^ |
| NAFLD (female)  (n=70) | 5.62(5.28,6.43) ^**^ | 10.03(8.87,11.71) ^**^ | 10.25(8.52,13.36) ^**^ | 7.97(6.42,11.45) ^**^ | 5.22(4.19,7.26) ^**^ |

Data are median (interquartile range).

^#^ *P* < 0.05, compared with the male Con group. ^##^ *P* < 0.01, compared with the male Con group.

^*^ *P* < 0.05, compared with the female Con group. ^**^ *P* < 0.01, compared with the female Con group.
